# Disorienting or Transforming? Using the Arts and Humanities to Foster Social Advocacy

**DOI:** 10.5334/pme.1213

**Published:** 2024-03-13

**Authors:** Snow Wangding, Lorelei Lingard, Paul Haidet, Benjamin Vipler, Javeed Sukhera, Tracy Moniz

**Affiliations:** 1 Department of Medicine, Schulich School of Medicine and Dentistry, Western University, London, Ontario, Canada; 2 Department of Medicine, Centre for Education Research and Innovation, Schulich School of Medicine and Dentistry, Western University, London, Ontario, Canada; 3 Woodward Center for Excellence in Health Sciences Education, Penn State College of Medicine, Hershey, Pennsylvania, USA; 4 Division of Hospital Medicine, University of Colorado Hospital, University of Colorado School of Medicine, Aurora, Colorado, USA; 5 Departments of Psychiatry, Hartford Hospital and the Institute of Living, Hartford, Connecticut, USA; 6 Department of Communication Studies, Mount Saint Vincent University, Halifax, Nova Scotia, Canada

## Abstract

**Introduction:**

The arts and humanities (AH) have transformative potential in medical education. Research suggests that AH-based pedagogies may facilitate both personal and professional transformation in medical learners, which may then further enhance the teaching and learning of social advocacy skills. However, the potential for such curricula to advance social advocacy training remains under-explored. Therefore, we sought to identify how AH may facilitate transformative learning of social advocacy in medical education.

**Methods:**

Building upon previous research, we conducted a critical narrative review seeking examples from the literature on how AH may promote transformative learning of social advocacy in North American medical education. Through a search of seven databases and MedEdPORTAL, we identified 11 articles and conducted both descriptive and interpretative analyses of their relation to key tenets of transformative learning, including: disorientation/dissonance, critical reflection, and discourse/dialogue.

**Results:**

We found that AH are used in varied ways to foster transformative learning in social advocacy. However, most approaches emphasize their use to elicit disorientation and dissonance; there is less evidence in the literature regarding how they may be of potential utility when applied to disorienting dilemma, critical reflection, and discourse/dialogue.

**Discussion:**

The tremendous potential of AH to foster transformative learning in social advocacy is constrained due to minimal attention to critical reflection and dialogue. Future research must consider how novel approaches that draw from AH may be used for more robust engagement with transformative learning tenets in medical education.

## Introduction

The arts and humanities (AH) occupy a pivotal position in medical education in furthering humanistic patient care [1,2,3]. Educators endeavour to teach and engrain attributes of compassion and patient-centred practice in their learners, which have been shown to benefit both individual patient health and the healthcare system [4,5,6]. Even so, there remain challenges with the integration, delivery, and assessment of the AH within medical curricula.

Numerous studies have elucidated the value of the AH in medical education, particularly their use in acquiring skills and fostering empathy. Dennhardt et al.’s seminal paper characterizes a framework delineating three epistemic functions of the AH in medical education: arts for mastering skills; arts for perspective taking, interaction, and relational aims; and arts for personal growth and activism [7]. Moniz et al. later develop the Prism Model, which provides a theory of practice largely indicating four functions of use for the AH in medical education. In the Prism Model, Dennhardt et al.’s third epistemic function of art for personal growth and activism is explored and split, distinguishing personal growth and activism as distinct functions. Furthermore, social advocacy is chosen in place of activism in reflecting the literature’s growing focus on social justice [8]. This is especially intriguing for medical education, as social advocacy is a critical component of the physician identity and growing increasingly important in a healthcare context [9,10]. Through stakeholder interviews, a recent exploration of the AH in medical education identified the need for healthcare providers to be socially conscious, and the corresponding need for education to address this gap in learner curricula [8]. While identified as a core value for medical learners, education surrounding social advocacy remains challenging.

A growing body of work suggests the value of transformative learning theory in medical education [11,12,13,14]. As characterized by Mezirow and developed in varying ways by theorists such as Freire and Hoggan, transformative learning describes a process in which the learner is fundamentally changed [15,16,17]. This occurs through a series of stages: a disorienting dilemma experienced by the learner, a period of critical reflection, the acceptance of new or different worldviews and, eventually, transformation into an agent of change or role adoption [16]. While transformative learning has been considered in the context of medical education in areas such as professional identity formation and bias mitigation [18], efforts toward social advocacy development using AH-based practices remain unclear.

An analysis of whether and how the AH are currently being used to facilitate transformative learning in the education of social advocacy skills has yet to be undertaken. Such a study would be worthwhile in informing how AH can be integrated in medical curricula to successfully educate learners and provide educators with a framework upon which to study and further pursue transformative learning. Thus, this study seeks to understand how the AH are being used in medical education curricula to facilitate transformative learning, specifically social advocacy.

## Methods

In 2019, Moniz et al. conducted a scoping review of the literature on the AH in North American medical education [19]. This endeavour generated a data set synthesized from a collective search of seven databases: PubMed, ERIC, CINAHL, Cochrane Library, Web of Science, PsycInfo, and EMBASE. The search parameters included English-only results published between 1991 to 2019 from the United States and Canada. Foundational works from programs outside these countries were also included. This study generated the Prism Model and outlined the literature’s predominant positioning of AH for perspective-taking and mastering skills. The stakeholder interview phase of this scoping review also emphasized the value of AH for social change and critique. Thus, while the aforementioned study identified social advocacy as an epistemic function of AH in medical education, we wanted to further explore *how* exactly the AH were being used for learning and whether the framework of transformative learning could guide the evaluation of this body of literature. Articles identified as using the AH to promote social advocacy (n = 27) in the generation of the Prism Model were analysed in the present study.

A search of MedEdPORTAL was conducted in 2021 with the aim of updating original findings and filling a pedagogical gap observed in texts obtained in the Prism Model, producing additional texts (n = 2). This search was expanded to 2017–2021 inclusive, as the period 2019–2021 generated one text from keyword searches. Search terms included: humanities, arts, social advocacy, transformative learning, transformation. Terms ‘transformative learning’ and ‘transformation’ were added to determine MedEdPORTAL’s proximity to transformative learning research and assess its contribution to the current study. Conflicts in our review process were arbitrated by group discussions over Zoom to reach majority consensus.

### Eligibility Criteria

Studies were included if they met criteria threefold for addressing social advocacy, AH, and transformative learning. Social advocacy criteria guided literature based on “social conscience and attention to systems of inequality, power, and privilege and working to eliminate social inequities and injustice in the interest of the common good” [8]. The basic stages of the transformative learning process sufficed for inclusion: disorienting dilemma, critical reflection, discourse/dialogue, including any explicit mention of the term ‘transformative learning’ or ‘transformation’.

### Analysis

Together, we collected descriptive data from included articles, such as study design, learner level, and teacher technique. We also included the duration of learning activities, for example if there was a single session or more of a longitudinal approach. As a group, once inclusion criteria of transformative learning was met, we further extracted and performed thematic analysis guided by Merriam and Bierema’s work on adult learning [20].

Drawing on theorists such as Freire, Kasworm, and Bowles, Merriam and Bierema posit Mezirow’s individual learning theory through a lens of social change and situate transformative learning in classroom and community settings. Freire’s work on social action is fundamental; wherein the transformation of oppressive social structures is accomplished through a process involving critical reflection and dialogue – moving a learner from passive acceptance to an agent of change. Merriam and Bierema further explore Kasworm and Bowles’ work in higher education, finding learning environments suitable catalysts for challenging students to move beyond comfort zones [20]. The depth of a text’s use of transformative learning was therefore assessed by its comparison to such descriptors in Merriam and Bierema’s text: disorienting dilemma (a significant life event precipitating transformative learning), critical reflection (confrontation of a learner’s original beliefs), and/or discourse/dialogue (discussion with others or self in hopes of building a new understanding) [20]. For example, an intervention in a classroom such as watching films on climate change would qualify as a disorienting dilemma depending on learner experience per Kasworm and Bowles, even though it may not be a significant life event such as the death of a loved one or involvement in random violence, per Mezirow’s early depictions. We characterized agent of change and role adoption together, as these last stages seemed to feed into one another during our search. In the process, we also noted if authors attempted to address *how* AH were being used to facilitate transformative learning of social advocacy, and if additional epistemological concepts were being used to guide this.

## Results

### Descriptive findings

Of the initial 27 texts identified in Moniz et al’s Prism Model study, nine texts met inclusion criteria, in addition to two publications from our search of MedEdPORTAL, forming 11 total texts. A diverse set of published work was included. Most texts were curricular reports, which often outlined a course’s syllabus, author reflection, and course outcomes. Following curricular reports were conceptual pieces [21,22], original research [23], and ‘other’ wherein the author provided a critical appraisal of film pieces [24]. The one empirical study used a qualitative methodology [23]. Curricular reports included courses in pre-medical education [25], undergraduate [26,27], and graduate medical education [28]. Studies also described courses traversing multiple learner groups and practicing health professionals. One such article described learning in both undergraduate and professional settings [29], while another explored both graduate and professional education [30]. A final curricular report described an experience for first-year learners from pre-medical, medical, and other health professional education [31].

In addition to the diversity of learner groups, the duration of learning experiences also varied from single episodic-like events to month-long recurring courses. Singular curricular interventions included play-reading, film-viewing, and verbatim theatre [24,30,31]. More longitudinal forms of learning occurred through diverse activities repeated over longer periods, such as engaging with visual works of art and group readings on texts concerning social and medical humanities over the course of a school term [26,29]. The length of academic terms also differed, with one text specifying a course occurring over seven weeks while another occurred over one month [28]. One paper also described its own development from a single event to an invited series [30]. Overall, the duration of interventions differed from short workshops to longer courses, indicating different methods of delivery in how the AH were facilitating transformative learning of social advocacy.

### The role of arts/humanities and the disorienting dilemma

Comparatively, more focus and curricular content were devoted to the facilitation of the disorienting dilemma, and we therefore directed our efforts to the use of AH in reference to the beginning of Mezirow’s transformative learning cascade. Experiences interpreted as disorienting dilemmas occurred both within the confines of a dedicated learning environment, such as classrooms or during clinical encounters, and externally in the community during service-based learning. Within classroom settings, learners were exposed to social themes of climate change, disability, and racism through various modalities of AH [24,26,29]. Learner engagement varied between studies, from studying texts surrounding specific social themes [26,29], to actively engaging in plays [23,30,31]. Studies incorporating play-like activities increased student engagement and created pseudo-scenarios capable of eliciting learner discomfort and initiating transformative learning [23,30,31]. In clinical settings, learners interacted with patients in ways that challenged existing worldviews and served to disorient and unsettle [25].

Some articles attempted to address how AH were used to facilitate social advocacy and transformative learning. Among curricular reports and empirical texts, the arts were construed as a prompt for eliciting the disorienting dilemma [23,24,27,30,31], or as instruments to process dissonance experienced by learners related to the disorienting dilemma [25,28], or integrated aspects of both [26,29]. Both conceptual papers outlined the use of AH as tools to facilitate the disorienting dilemma through overlapping theories [21,22]. The first of these is the theory of estrangement. In a paper by Kumagai and Wear, authors traced the intersection of Shklovsky’s estrangement and Brecht’s alienation effect in medical education through a theory of making strange, in which “art forces its viewers to look on the familiar in new ways—ways from which new ways of seeing, thinking, and being arise” [21]. Such language is reminiscent of Mezirow’s transformative learning, with art being the catalyst or initiator of the learning process. In a separate paper by Bleakley, the purpose of art was identified as “consciousness-raising through creating discomfort, challenge or ambiguity” [22]. In such conceptual terms, AH were used to evoke the disorienting feelings and changes in perception associated with transformative learning. In addition to serving as an elicitation prompt, Bleakley also described the role of AH as part of a three-step process of ‘consciousness-raising’ which involves: first, a disruption to challenge habit; second, patterns of resistance emerging; and third, analysis of resistance to develop new awareness [22]. Thus, AH are shown to not only precipitate dissonance, but also provide a mechanism to process, evaluate, and reconstitute after disorientation.

The application of such theories is apparent in empirical and curricular reports. For example, Matharu observed the empirical use of play-reading a theatre script in approaching biases of obesity, thus creating the environment and introduction to the disorienting dilemma [23]. Jones also demonstrated this use of the arts, broaching topics of climate change through documentaries, photographs, and visual art, essentially introducing students to “enormous and catastrophic” issues wherein students are often “left feeling helpless” at the beginning of their learning [29]. In this piece on visual activism, visual images were used to create “defamiliarized and discomforting moment[s]” for learners in reference to seeing the concealment of disability [29]. Moreover, this pattern was evident throughout the literature, as AH-based activities confronted and unsettled learners by setting forth the foundational stage of transformative learning. A service-learning course showed particularly close integration of the humanities throughout the education process. In this course, literature and poetry were used to both introduce and enhance topics of ethics, illness experiences, and other medically relevant subjects as students shadowed physicians and volunteered in the community [25].

While eliciting a disorienting dilemma was the primary focus of this body of work, the theory of transformative learning itself was described inconsistently. Some pieces directly named Mezirow’s process, while others featured stages of the learning theory distinct from the whole. A curricular report on verbatim theatre described transformative learning and its basis for use with the creative arts, reading, “creative arts–based approaches with a social component have the potential to provide a disorienting dilemma seen as foundational to transformative learning” [31]. Delving further, this piece explored reflection, such that “reflective writing enhances the potential for transformative, deeper learning” [31]. Here, transformative learning was used pedagogically to guide curricular development and learning. In Kumagai and Wear’s piece, the disorienting dilemma was directly attributed to Mezirow [21]. Other texts were less explicit in naming the pedagogical process, yet alluded to the theory through terms such as “transformative potential” [25].

### Variation in how critical reflection is applied in practice

We primarily used Mezirow’s work on critical reflection to guide our inclusion of texts describing aspects of reflection. Mezirow outlined a critique of learner presuppositions, essentially if learners questioned their deeply held assumptions of their own experiences and the world around them [16]. As a result, we found that reflection was less frequently cited throughout this body of work, and the depth of learner reflection was mixed. Furthermore, it was difficult to discern whether ‘critical’ reflection occurred – that is, if learners confronted or critiqued the beliefs upon which their fundamental suppositions were developed. For example, one article outlined the use of a resident passport, essentially a reflective writing journal with 21 guided questions [28]. Reflection was promoted using reflection feedback loops, in which assessing faculty directly requested additions or revisions to entries. This level of reflection was sustained by smaller, monthly reflective pieces following completion of the passport. While rich with activities endeavouring to facilitate reflection, from the exemplar journal entries it was unclear if learners in fact achieved the ‘critical’ aspect of reflection. For example, assignments included prompts for reflecting on experiences of being stereotyped and describing conflicting beliefs with patients affecting patient care. Yet provided reflections lacked sufficient depth, with entries namely pointing to changes to be made in future patient interactions, with little detail regarding self-reflection or consideration of the writer’s prior beliefs [28]. Therefore, while reflection itself was clear, it was unclear whether these activities met the level of critical reflection to sustain and drive transformative learning.

Another study described reflection facilitated through group discussions yet lacked detail regarding efforts of continuation or repetition, and whether reflection drove specific change in learners. Consider the following excerpt from this study:

After the reading, all students present discussed the play among themselves, with minimal nondirective facilitation by the study coordinators. Discussion often centered upon the hardships encountered by the main characters. Frequently, students recalled personal experiences of loved ones who were overweight that mirrored what happened to characters in the play. Those who read a part often reflected on their character, either understanding or confronting their perspective, which may have conflicted with the student’s expectations [23].

While reflection is evident in this example, it is unclear if this reflection prompted learners to action, thus enabling the continuation of transformative learning.

This is not to say that critical reflection was altogether absent in our search. In fact, one article described the use of service learning, which actively incorporated community service experiences with critical reflection. Excerpts from student reflection journals demonstrated insight into the learner experience, with described observations, lessons, and challenges to individual worldviews [25]. For example:

“I felt foolish for believing I was so different from them. …I learned more about the independence of disabled people: most packed and brought their own lunch, others were excellent at sewing, and almost all of them had superb communication skills. I was actually ashamed that I stereotypically believed that these people were so different [from] me” [25].

Overall, however, we found ambiguity in the presence of critical reflection and further inconsistency in how critically reflective activities were used in the pursuit of transformative learning.

## Discussion

We found in the literature an overwhelming focus on Mezirow’s disorienting dilemma compared to critical reflection and discourse/dialogue, which suggests a spotlight on content over pedagogy. AH therefore appear to spark a disorienting dilemma in initiating transformative learning, but educational endeavours lack pedagogic follow-through once these disorienting dilemmas occur.

### Content over pedagogy

During our analysis, we found a dearth of examples of how authors were leveraging AH for pedagogical purposes. Much of the existing literature provided descriptive content on the AH. For example, in discussing concepts of estrangement in media, the author dedicates much of the text’s space to descriptions and analyses of videos, concluding with a film’s use in small group discussions [24]. While showcasing the application of video forms on social advocacy, learner engagement is not much explored. Instead, discussion questions are provided with little sense of learner experience or development. In our initial inquiry, we found this to be a prevalent issue; educators focused on describing their AH content rather than the pedagogical use of said content for transformative learning.

Among others, current literature suggests several reasons for this paucity of pedagogy, including learner perceptions of utility [32], traditional biomedical vs. biopsychosocial models of practice [33], and methodologic challenges in assessing transformative learning [34]. An intriguing proposal for this focus on content over pedagogy lies in the absorbing nature of the AH themselves. For the same reason AH have been used successfully to engage learners, educators are immersed in the subject of their studies, unintentionally foregoing pedagogical focus. In fact, this observation is not unique to the field of medical education. AH have been used in several curricula, helping students grasp concepts across the sciences, judicial studies, mathematics, and more [35,36,37,38,39,40]. Where these methods have allowed for enriched learning experiences and sparked classroom interest, educators are themselves likewise engaged by studies of film, literature, and varying modalities of art [41,42].

Yet high levels of engagement may also be problematic. On behalf of learners, there is the risk that initial engagement does not translate to the overall learning process [43,44,45]. Present studies demonstrate the positive relationship between teacher enjoyment in their field of education and student experience [46,47,48]. Despite this, an exceeding focus or unintentional preoccupation of their own subject of study may obscure learning and detract from the educational role of their tools. There lacks a body of literature exploring the negative effects of educator enjoyment; however, such consequences may be suggested in our findings as well as reflective pieces from educators [24,49]. Interestingly, educators may undergo an element of their own transformative learning through this process, which may complicate the pedagogical process by way of role conflict [50]. There therefore strikes a tenuous balance between the educator and learner’s experience in the use AH for pedagogy.

### Privileging disorientation

Amongst these texts, facilitators and educators were selective in their application of the dimensions of transformative learning. In particular, the ‘disorienting dilemma’ was most prominent, while other aspects such as critical reflection and role adoption were largely overlooked. Other scholars have noted this pattern: a recent study found substantial use for transformative learning without employing each of the core tenets of Mezirow’s theory, particularly finding a lack of use for rational discourse [51]. This selectivity may have critical consequences: by taking on fragments of the theory, the established results may also be fragmented, rendering a half-hearted understanding of how transformative learning may be used with the creative AH. This may indicate that some aspects of Mezirow’s theory are required to generate sufficient transformation, such as the disorienting dilemma and critical reflection, whereas a higher threshold of other tenets may be needed for similar results. Just as educators have distilled core tenets from Mezirow’s original 10-step process [20], it is likely that further refinement of this theory will be observed in the educational landscape with risks of diluting learner outcomes.

### Learning through dissonance

We found several theories framed alongside transformative learning in contextualising a larger body of dissonance theory work. Named amongst theorists such as Dewey, Freire, Shön, and Habermas, Mezirow is well positioned within their respective pedagogies of dissonance/estrangement. We namely saw elements of Dewey’s forked road invoked in our texts, in which a dilemma proposes alternative paths of uncertainty and ambiguity, thus inciting a learner into action. In Jones’ work on visual activism, when learners encountered films and images on concealed and visible disability, they were provided with an uncomfortable opportunity to consider the biomedical and social models of disability, urging them to “recognize and apprehend a newness that can be transformative” [29]. Such tasks draw on both Mezirow’s disorienting dilemma in creating the disruptive experience and Dewey’s alternative paths in producing such discomfort. Consideration of this existing body of dissonance learning theory is necessary in evaluating the use of transformative learning, as it appears as one component of the larger, overlapping whole.

## Limitations

This project was formed from the basis of a previous scoping review by Moniz et al, which provided the database for our search. As a result, further searches outside of the aforementioned database and MedEdPORTAL were not performed. This will have shaped both our sample and our primary results. From searches of MedEdPORTAL, there is a preliminary sense that including texts from this source in a future review may extend insights into how educators are approaching the pedagogy. Additionally, as Moniz et al.’s scoping review was published in 2021, our findings may not reflect the most recent landscape of the arts and humanities and medical education literature, particularly in the setting of a post-pandemic social change. While we intermittently searched MedEdPORTAL in recognition of this, some texts may have been excluded between the initiation of our project and final phase of data extraction.

## Conclusion

Our narrative review presents insight into how the AH are being used to facilitate transformative learning and social advocacy in medical curricula. Namely, we found evidence for AH practices such as verbatim theatre, play-acting, film-viewing, and other methods for facilitating Mezirow’s transformative learning. Such results indicate an unexplored depth of potential for AH. Yet we also found aspects of literature lacking, with AH social advocacy-based studies falling short of full transformative learning by focusing almost entirely on the disorienting dilemma. Future research and applications of transformative learning must consider how novel approaches drawing from AH may foster discussion and action.
